# Two lymphoma histotypes and papillary thyroid carcinoma coexisting on Hashimoto ground: a case report and review of the literature

**DOI:** 10.1186/s13000-024-01472-7

**Published:** 2024-03-09

**Authors:** Igor Iskra, Maja Ilić Tomaš, Tatjana Bogović Crnčić, Edvin Kukić, Ita Hadžisejdić, Manuela Avirović, Neva Girotto

**Affiliations:** 1 Clinical Department of Nuclear Medicine, Clinical Hospital Centre Rijeka, Rijeka, Croatia; 2 Clinical Department of Pathology and Cytology, Clinical Hospital Center Rijeka, Rijeka, Croatia; 3 Faculty of Medicine, University of Rijeka, Rijeka, Croatia

**Keywords:** Papillary thyroid carcinoma, Primary thyroid lymphoma, Diffuse large B-cell lymphoma, Mucosa-associated lymphoid tissue lymphoma, Hashimoto thyroiditis

## Abstract

**Background:**

Papillary carcinoma is the most frequent type of thyroid carcinoma, while primary thyroid lymphoma is uncommon disease. The coexistence of these entities has already been described, and the common risk factor is considered Hashimoto thyroiditis. The two most frequent histotypes of primary thyroid lymphoma are diffuse large B-cell and mucosa-associated lymphoid tissue lymphoma, but the coexistence of both with papillary carcinoma is rarely reported.

**Methods:**

We present a case of a previously healthy 57-years old male with rapidly growing lump on the right side of the neck. Ultrasonography revealed nodules in both thyroid lobes. Fine needle aspiration cytology and pertechnetate scintigraphy were performed. Due to the Bethesda T-5 in the “cold” nodule of the right lobe, surgery with histopathological and immunohistochemistry analysis was indicated.

**Results:**

Histopathological and immunohistochemistry methods confirmed concomitant malignancies in the thyroid gland: diffuse large B-cell lymphoma and papillary carcinoma in the right, and mucosa-associated lymphoid tissue lymphoma in the left lobe with Hashimoto thyroiditis in the remaining tissue. Patient underwent therapy procedures and was without signs of local recurrence or metastatic spread on subsequent follow-up.

**Conclusions:**

Sudden appearance of the neck mass in patients with Hashimoto thyroiditis should raise suspicion on primary thyroid lymphoma and be promptly taken in the diagnostic workup, including fine needle aspiration cytology. Pathology with immunohistochemistry is crucial for further clinical decision making. Since the standardized protocol in management of these complex patients is missing, personal approach and close collaboration between cytologist, pathologist, surgeon, haematologist and nuclear medicine specialist is essential.

## Introduction

Primary thyroid lymphoma (PTL) is a rare condition and accounts for 1 to 5% of all thyroid malignancies and up to 7% of all extra nodal lymphomas [1–4]. It usually originates from B cell lineage and is more frequent in older women [3, 5]. The two most common histotypes are diffuse large B-cell lymphoma (DLBCL), present in 50 to 70% of all primary Non-Hodgkin thyroid lymphomas and mucosa-associated lymphoid tissue lymphoma (MALT), present in 10 to 50% [3, 5]. Papillary thyroid carcinoma (PTC) is the most frequent type of endocrine thyroid carcinoma, and accounts for 70% of all thyroid malignancies with rising incidence in the past decades mainly due to incidentally discovered microcarcinoma [6]. The diagnostics of thyroid lesions in general includes thyroid ultrasonography (US), fine needle aspiration cytology (FNAC) and ^99m^Tc pertechnetate scintigraphy, but sometimes, especially in the case of PTL, the diagnosis is not straight forward. Clinically, both entities can be characterised with solitary neck nodule appearance, but usually with different growth dynamics and symptoms. In a case of rapid nodule growth, anaplastic carcinoma must also be considered. The most important risk factor for PTL is chronic autoimmune lymphocytic thyroiditis (Hashimoto thyroiditis), associated with more than 90% of PTL [7, 8]. Hashimoto thyroiditis (HT) has also been considered a risk factor for papillary thyroid carcinoma development, but not for the other thyroid cancer types [9–11]. The coexistence of PTL and PTC is rare and the presence of two simultaneous PTL histotypes, MALT and DLBCL with PTC has been described only in few cases so far. Therefore, we report the case of these malignancies coexisting with Hashimoto thyroiditis.

## Case report

We report a case of a 57-year old male patient without pre-existing health condition who presented in July 2018 with rapidly growing lump on the right side of the neck he had noticed four days prior to the referral, accompanied with fatigue and local irritation. A written informed consent was obtained from the patient.

**Fig. 1:**
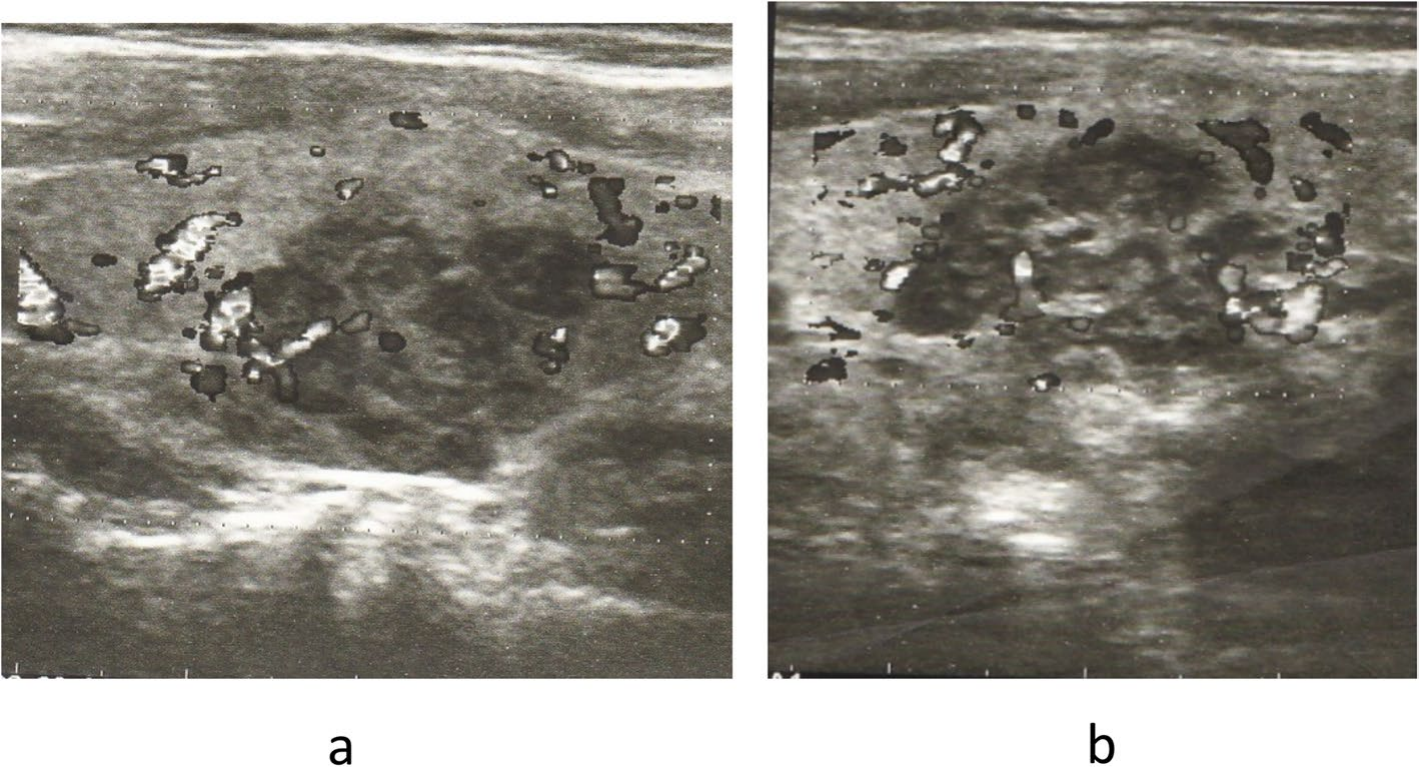
**a** Thyroid ultrasound. Right thyroid lobe with hypoechoic, perinodally vascularized nodule measuring 30 × 25 × 36 mm. **b** Thyroid ultrasound. Left thyroid lobe with hypervascularized, irregular hypoechoic area measuring 20 × 12 × 16 mm

**Fig. 2:**
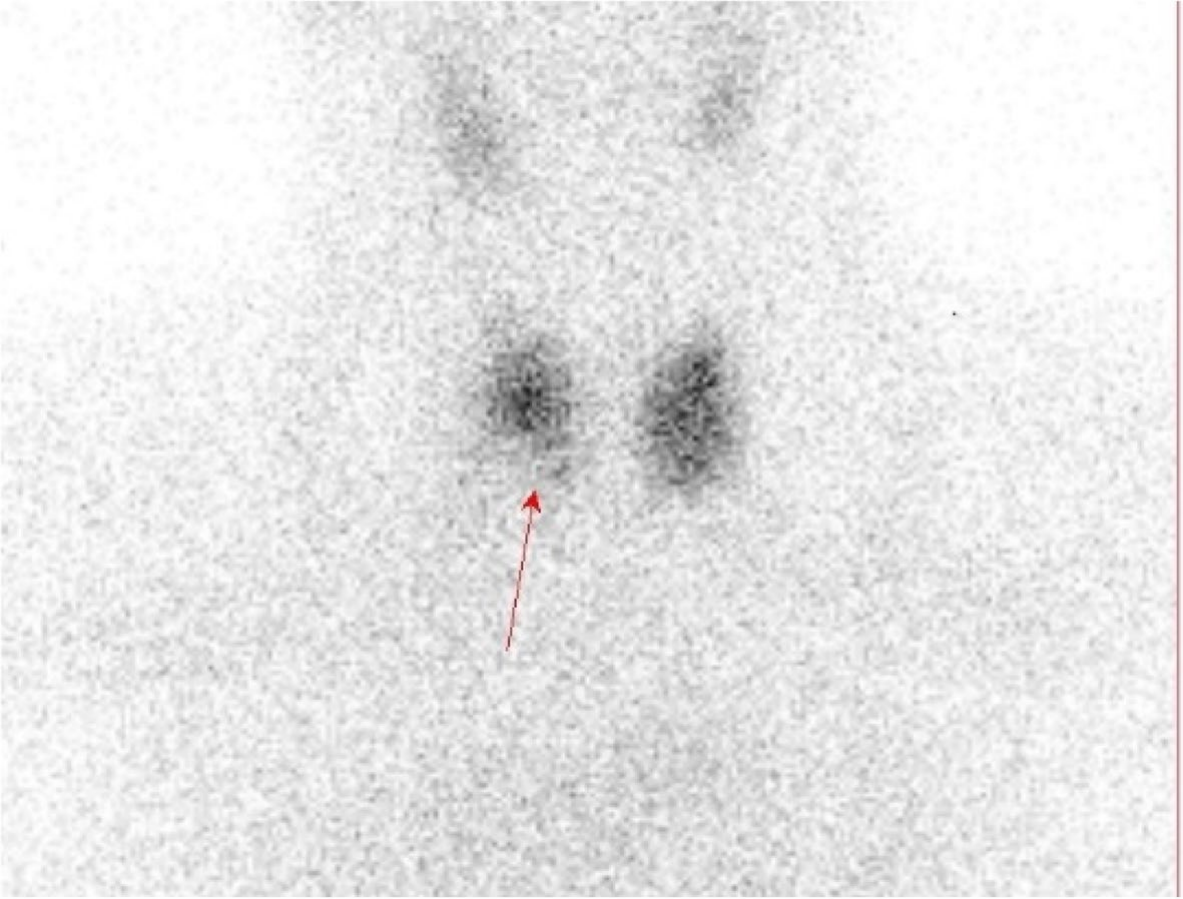
Thyroid ^99m^Tc pertechnetate scintigraphy showing cold area in the right thyroid lobe indicated by arrow

Clinical exam confirmed palpable, large nodule on the right side of the neck. Thyroid hormone and thyrotropin (TSH) levels were within the normal range (free triiodothyronine 6.82 pmol/L, free thyroxine 14.2 pmol/L and TSH 4.97 mIU/L). Thyroid antibody titres were also normal (thyroglobulin antibodies < 20.0 IU/mL and thyroid peroxidase < 10.0 IU/mL). Neck ultrasonography revealed a hypoechoic, perinodally vascularized nodule in the right thyroid lobe measuring 30 × 25 × 36 mm (EU – TIRADS 4), and hypervascularized, irregular hypoechoic area in the left thyroid lobe measuring 20 × 12 × 16 mm (EU – TIRADS 5) (Fig. 1). Thyroid scintigraphy with ^99m^Tc-pertechnetate was also performed showing “cold” area in the right thyroid lobe, while the left was unremarkable (Fig. 2). Following fine needle aspiration cytology, both changes were classified, the one in the right lobe as Bethesda T-5, and in the left lobe as Bethesda T-2, combined with florid lymphocytic inflammation.

Patient was subsequently examined by a haematologist, endocrinologist and otolaryngologist who did not report on any additional significant clinical findings. Routine blood tests, protein electrophoresis and cytological analysis of peripheral blood smear were unremarkable, except for the higher erythrocyte sedimentation rate (ESR = 66) and slight anisocytosis.

**Fig. 3:**
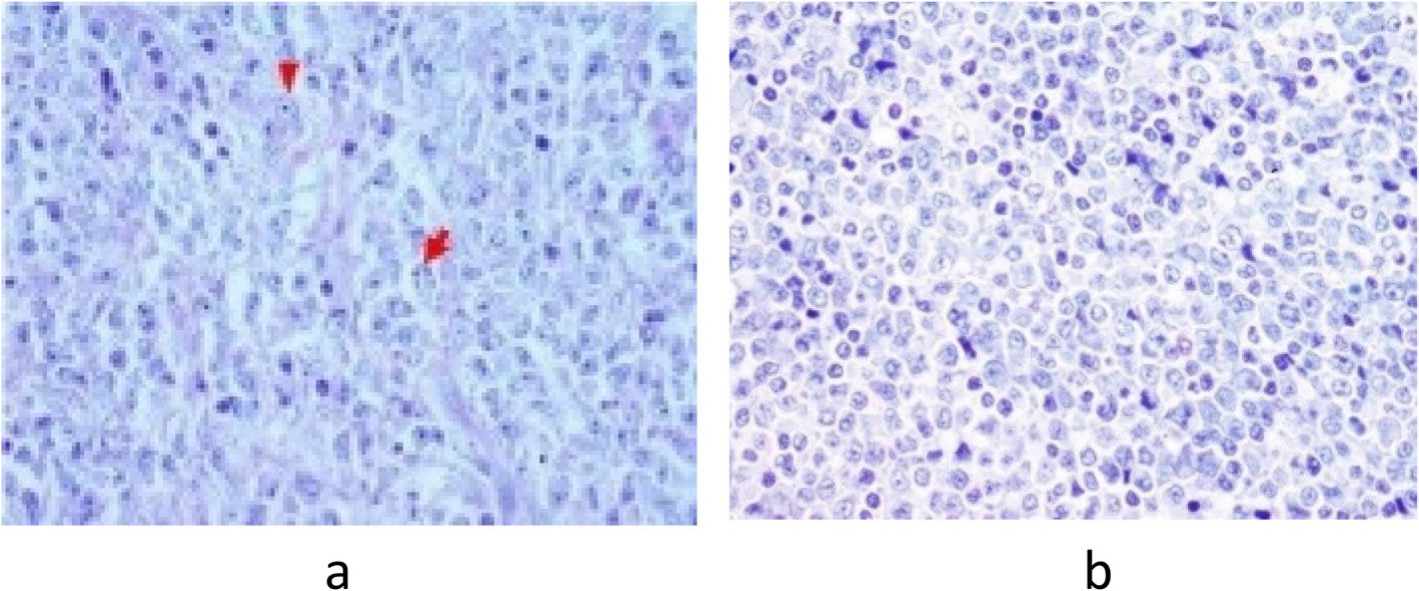
**a** DLBCL. Diffuse proliferation of medium sized to large lymphoid cells with vesicular nuclei with 2–4 nuclear, membrane-bound nucleoli (centroblasts—red arrows) or immunoblasts (large cells with single, central nucleolus) (Giemsa, magnification 40x). **b** MALT lymphoma. Predominate medium sized lymphocytes with round nuclei resembling those of centrocytes, in between are scattered large transformed cells (Giemsa, magnification 40x)

**Fig. 4:**
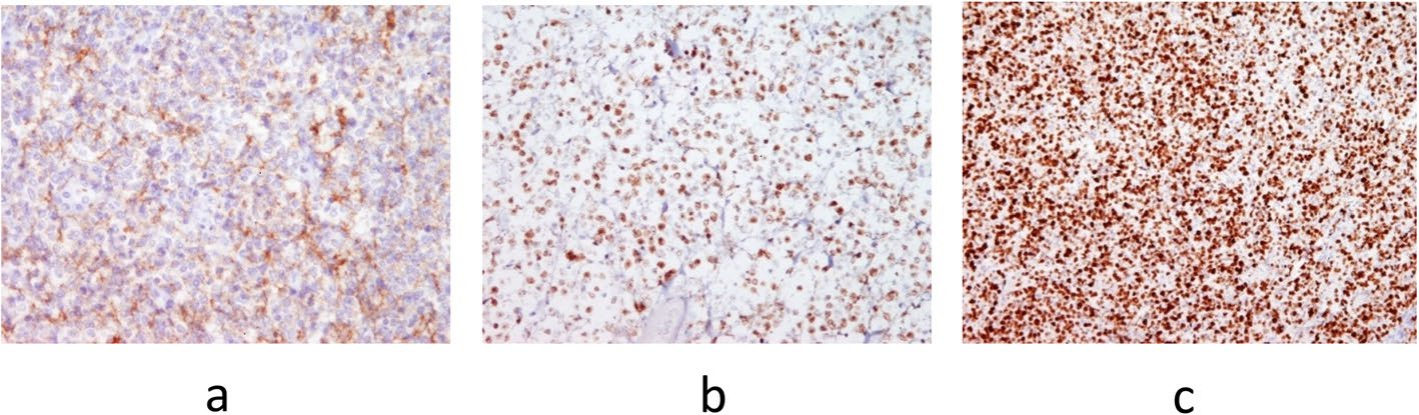
**a**-**c** Immunohistochemical staining of DLBCL area. The majority of the cells are immunohistochemically negative for CD10 (A, magnification 20x), while Bcl-6 staining is positive (B, magnification 20x) with very high Ki-67 proliferation index (C, magnification 10x)

**Fig. 5:**
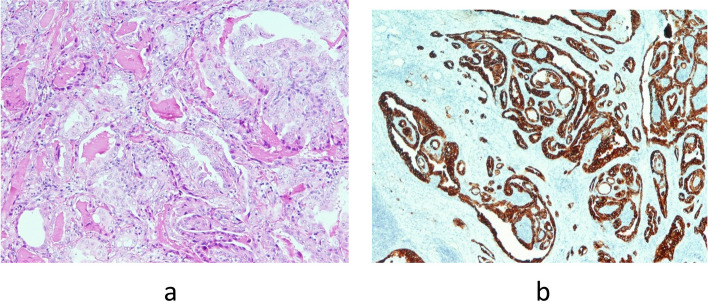
**a** Tumour tissue is composed mainly of follicles lined by layer of cells showing nuclear characteristics of the papillary thyroid carcinoma (HE, magnification 100x). **b** Strong and diffuse cytoplasmic immunohistochemical staining with anti CK19 confirms papillary thyroid carcinoma (CK19, magnification 100x)

**Fig. 6:**
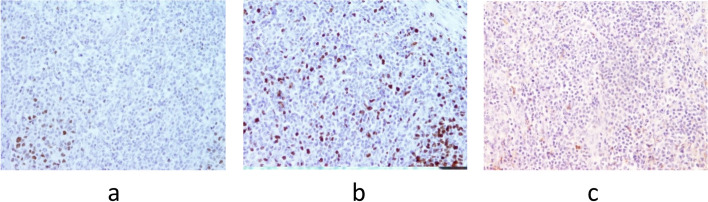
**a**-**c** Immunohistochemical staining of extranodal marginal zone lymphoma (MALT-oma). CD10 (A) and Bcl-6 (B) staining is negative with low Ki-67 proliferation index (C); in the image B and C recognizable germinal centers which are Bcl-6 positive and have high Ki-67 proliferative index (A-C, magnification 20x) can be seen

Control ultrasonography of the neck region was performed after eight days along with ultrasonography of the abdomen and both inguinal and axillar regions, confirming one hypoechoic zone in each thyroid lobe. All regional lymph nodes were characterised as unremarkable, and abdominal ultrasound was normal. Repeated FNAC of the right lobe nodule confirmed Bethesda T-5 with a suspicion of possible lymphoproliferative disease. Therefore, patient was discussed with the Multidisciplinary team for thyroid diseases, where the right thyroid lobectomy was suggested. Initially, one month from the first visit (beginning of August), right thyroid lobectomy was performed and a nodule of a greyish cutting surface and medium hard consistency, but centrally softer was described. Pathology results showed specific characteristics of DLBCL of non-germinal center origin—a diffuse proliferation of medium to large sized lymphoid cells with vesicular nuclei containing centroblasts or immunoblasts (Fig. 3a). Majority of cells were immunohistochemically negative for CD10 (Fig. 4a) while Bcl-6 staining was positive (Fig. 4b) with very high Ki-67 proliferation index (Fig. 4c). Also, the histopathological analysis reported on the small papillary thyroid carcinoma (11 × 8 mm) in the remaining tissue of the right lobe, adjacent to DLBCL lesion. The tumour was composed mainly of follicles lined by a layer of cells with nuclear characteristics of papillary thyroid carcinoma with strong and diffuse cytoplasmic immunohistochemical staining with anti CK19 (Fig. 5a and b). Left thyroid lobectomy was subsequently performed and a tumour nodule of a white—greyish colour was described. Final pathology of the nodule in the left lobe returned as MALT lymphoma containing medium sized lymphocytes with round nuclei resembling those of centrocytes (Fig. 3b), immunohistochemically negative for CD10 (Fig. 6a) and Bcl-6 (Fig. 6b) with low Ki-67 proliferation index (Fig. 6c). There was only a slightly higher number of blast-type cells in places, but without the clear formation of larger clusters and transformation into DLBCL. Chronic, Hashimoto thyroiditis was found in the remaining thyroid tissue.

Whole-body Fluorine – 18 fluorodeoxyglucose positron emission tomography/computed tomography (^18^F-FDG PET/CT) scan, bone marrow biopsy and cytological analysis of peripheral blood smear were performed subsequently, showing no clear evidence of metastatic spread, except some nonspecific ^18^F-FDG accumulation in the neck region (standard uptake value, SUV 3.2). The Onco-haematological team indicated four-cycles of immune chemotherapy with Rituximab, Cyclophosphamide, Hydroxydaunorubicin hydrochloride, Vincristine and Prednisone (R-CHOP protocol), followed by radiotherapy and second ^18^F-FDG PET/CT scan upon completion of the therapy. The second ^18^F-FDG PET/CT was unremarkable and without accumulation in the neck region, suggesting previously detected ^18^F-FDG positive region as transitory postoperative inflammation. Conformal radiotherapy of the neck (3000 cGy, 15 fractions) was performed in the following months. In the meantime, after total thyroidectomy, patient was started on levothyroxine hormone supplementation (100 µg per day) with occasional dosage adjustments at routine controls which included thyroglobulin, thyroglobulin antibodies measurements and neck ultrasound exams. Six months after the completion of radiotherapy, and three weeks after thyroid supplementation withdrawal, radioiodine (RAI) ablation with 3700 MBq Iodine—131 was applied, followed by post-therapy whole-body scintigraphy showing only minor residual neck uptake in thyroid remnant, without evidence of iodine accumulation elsewhere in the body. On thyroid hormone supplementation of 200 µg of levothyroxine per day, regular haematological visits and visits to nuclear medicine department, the last in October 2023, five years from the diagnosis, the patient is considered disease free.

## Discussion

Primary thyroid lymphoma is a rare disease, more frequently presenting as DLBCL (50 – 80%), and only in up to 30% as MALT lymphoma. The third, follicular lymphoma accounts for only 12% of PTL [12]. The pathogenesis of PTL is not clear, but an increasing risk for lymphoma development within autoimmune disorder setting has been reported [12]. It generally presents as a rapidly growing neck mass, accompanied with compressive signs, with or without B-symptoms. In approximately 10% of cases it can also be accompanied with hypothyroidism [12].

Since it is known that DLBCL might develop from MALT lymphoma, simultaneous presence of both entities is also possible, although very rare [13].

Primary thyroid lymphoma appearance has been associated with papillary thyroid carcinoma, which is the most frequent form of thyroid cancer, and accounts for 70% of all thyroid malignancies. It usually presents as a relatively well defined, slowly progressing solitary neck nodule, often incidentally found.

Coexistence of PTC with one of the two lymphoma histotypes has been described, more frequently with MALT [2, 14].

Both entities, PTL and PTC are associated with chronic autoimmune thyroiditis background, but the pathophysiological relation is not completely clear. According to some authors, Hashimoto thyroiditis is considered the most important risk factor for PTL appearance and a moderate risk factor for the papillary thyroid carcinoma [2, 3]. The connection between Graves´ disease and PTL has also been described [15]. It seems that chronic antigenic stimulation of lymphocytes might have a role in DLBCL growth, while chronic inflammation can induce synthesis of cyclooxigenase-2, present in both, Hashimoto thyroiditis and thyroid carcinoma [13]. On the other hand, there has been evidence that special immunophenotype of lymphocytes involved in the pathophysiology of autoimmune thyroiditis could be related to a higher antitumor response and consequently attenuate the progression of papillary thyroid carcinoma [16]. Nevertheless, simultaneous appearance of PTL, PTC and HT is rather rare and we have managed to found only 16 published cases reported in the last 15 years comprising total of 19 patients, all presented in Table 1 [2, 13, 14, 17–29]. The patients were predominantly women (12/19), mean age 57y, and in majority of cases PTL was of MALT type (*N* = 11). In four patients DLBCL was diagnosed, in one patient the thyroid lymphoma type was not clearly stated and in one patient other lymphatic pathology—chronic lymphocytic leukemia/small lymphocytic lymphoma was reported. Only in two patients simultaneous presence of both types, MALT and DLBCL was described [22, 28].

**Table 1:** The summary of previously reported cases of the coexistence of papillary thyroid carcinoma, primary thyroid lymphoma and Hashimoto thyroiditis

Series	Sex/age(y)	Presentation	Thyroid ultrasound	Thyroid function	Preoperative diagnosis (FNAC)	Pathologic diagnosis	Therapy	Follow-up
Hasan et al. 2014 [17]	F/60	Painless lump on the right side of throat for 3 months	Enlarged right lobe, heterogeneous parenchyma	Data not available	Non-diagnostic, possible lymphoproliferative disease or thyroiditis	MALT lymphoma, HT and PTC, follicular variant	Right thyroid lobectomy, radiation therapy	No recurrence for 3 years
Jayaprakash et al. 2014 [18]	F/32	A sudden increase of a solitary nodule for 2 weeks	Enlarged nodular thyroid with hyperechoic areas	Hypothyroidism	Features of HT and PTC	HT, small focus of PTC—follicular variant and NHL	Total thyroidectomy, L-thyroxine, chemotherapy	No recurrence for 1 year
Cheng et al 2012 [19]	M/59	Progressive thyroid enlargement for a few months, intermittent dysphagia	Discrete hypoechoic nodules in each lobe with speckled calcifications	Hypothyroidism	Right-sided nodule: predominance of associated lymphoid cells, possible low grade lymphoproliferative disorder. Left sided nodule: nuclear features of PTC	MALT lymphoma in the right lobe and PTC in the left, HT	Total thyroidectomy, L-thyroxine, RAI therapy	No recurrence for 6 years
Vassilatou et al. 2011 [20]	Case 1: F/51	Palpable nodule in the right lobe, palpitations	Multinodular goiter	Subclinical hyperthyroidism, anti -TPO +	Larger nodule in the right lobe – PTC, larger nodule in the left lobe—HT	PTC and Warthin-like papillary carcinoma in the right lobe, MALT lymphoma in the left lobe and extensive HT	Total thyroidectomy, RAI therapy	No recurrence for 1 year
	Case 2: M/63	Incidentally found small multinodular goiter on a carotid US	Multinodular goiter, larger nodule in the right lobe, hypoechoic	Normal	Right-sided nodule suspicious for malignancy	Invasive follicular carcinoma in the right lobe, PTC in the left, both sided HT, chronic lymphocytic leukaemia/small lymphocytic lymphoma	Total thyroidectomy, L-thyroxine therapy, RAI therapy	No recurrence for 1 year
De Melo et al. 2010 [21]	M/61	Painless thyroid enlargement for three months	Multinodular goiter, some nodules with gross calcifications	Normal	Not performed	Multicentric PTC, HT and MALT lymphoma	Total thyroidectomy, L-thyroxine, RAI therapy	No recurrence for 2 years
Alvarez -Vazquez et al. 2007 [22]	F/84	Enlarging thyroid mass for a month, dysphagia, stridor, hoarseness	Large goiter with bilateral jugular lymphadenopathy	Normal, anti-TPO +	Not performed	MALT lymphoma with focal transformation in DLBCL (extrathyroid extension), PTC – tall cell, HT	Total thyroidectomy, L-thyroxine, palliative external radiotherapy	Patient died 6 months later
Nam et al. 2013 [23]	F/81	Goiter, hoarseness and weight loss	Enlargement of the thyroid gland, both sided nodules	Normal	Suspicious for PTC	PTC in the right lobe (minimal extrathyroidal extension) and HT. MALT lymphoma in the left lobe	Total thyroidectomy, right and left central neck node dissection, L-thyroxine	No recurrence for 1 years
Levy-Blitchtein et al. 2016 [23]	M/54	Goiter enlargement for 9 months, cervical pain, dysphonia and dysphagia	Hypoechoic nodules in both lobes	Normal	Not performed	PTC (classic variant), extranodal MALT lymphoma, HT	Total thyroidectomy, L-thyroxine, RAI therapy	Not reported
Chen et al. 2019 [13]	F/37	Expanding neck mass for 4 weeks, dyspnea, dysphagia	Enlargement of the right thyroid lobe	Data not available	Data not available	DLBCL, PTC, HT- not clearly stated	Right thyroid lobectomy, chemotherapy	No recurrence for 1 year
Shen et al. 2015 [25]	F/25	Incidentally found small multinodular goiter on carotid US	Multinodular goiter; largest hypoechoic nodule in the right lobe	Normal	Atypical follicular epithelial cells and atypical lymphoid cells in the largest nodule	PTC, MALT lymphoma, HT	Total thyroidectomy, L-thyroxine, RAI therapy, chemotherapy	No recurrence for 2 years
Trovato et al. 2017 [26]	F/66	Enlargement of the right side of the neck, intermittent dysphagia	Hypoechoic nodule in the right lobe	Hypothyroidism	Atypical epithelial cells and lymphocytic infiltration	DLBCL, PTC (microcarcinoma, classic variant), HT	Total thyroidectomy, chemotherapy, RAI therapy	No recurrence for 2 years
Kir et al. 2018 [27]	F/77	Progressively enlarging thyroid gland for 2 years	Diffuse thyroid enlargement with heterogeneous nodularity	Normal	High-grade NHL and HT	DLBCL, HT, microscopic PTC	Total thyroidectomy, chemotherapy and RAI therapy	No recurrence for 2 years
Duger et al. 2020 [28]	F/65	Palpable nodules in the thyroid gland	Solid, hypoechoic nodules in both lobes	Normal	Benign findings of both nodules	PTC (microcarcinoma) in both lobes, MALT lymphoma, and DLBCL, HT	Total thyroidectomy	Not reported
Lan et al. 2018 [29]	Case 1: M/57	Enlargement and palpable nodules in both thyroid lobes	Multinodular goiter	Normal (anti-TPO +)	Not performed	PTC, MALT lymphoma, HT	Total left and partial right lobectomy, regional lymph node dissection, L-thyroxine	No recurrence for 5 years
	Case 2: F/43	No symptoms	Small calcified nodule in the isthmus. Thyroid enlargement	Hypothyroidism (anti-TPO +)	PTC	PTC, MALT lymphoma, HT	Total thyroidectomy	No recurrence for 5 years
	Case 3: F/61	Progressive enlargement of the thyroid gland	Hypoechoic mass in the right lobe, nodule in the left lobe	Normal (anti-TPO +)	Not performed	PTC, MALT lymphoma, HT	Right thyroid lobectomy radiotherapy	No recurrence for 5 years
Xie et al. 2015 [2]	Male/41	Painless left sided thyroid enlargement for 2 months	Hypoechoic nodularity in isthmus and left lobe	Normal (anti-TPO +)	Not performed	PTC, DLBCL, HT	Left thyroid lobectomy and isthmectomy, dissection of left cervical lymph nodes, chemotherapy, radiotherapy	No recurrence for 2 months
Whitehouse et al. 2020 [14]	M/83	Swelling of the right side of the neck	Enlarged right lobe, hyperechoic, partially calcified nodule	Hypothyroidism	Features of PTC, background inflammatory cells	PTC, MALT lymphoma, HT	Total thyroidectomy	No recurrence for 5 years

*Abbreviations*: *FNAC* Fine needle aspiration cytology, *HT* Hashimoto thyroiditis, *PTC* Papillary thyroid carcinoma, *MALT* Mucosa-associated lymphoid tissue, *DLBCL* Diffuse large B-cell lymphoma, *RIT* Radioactive iodine treatment, *anti-TPO* Autoantibodies against thyroid peroxidase, *US* Ultrasound

As showed in Table 1 and in accordance with literature, PTL and PTC have been more frequently found in middle aged and older women usually with long standing Hashimoto thyroiditis [1]. On the contrary, our patient was euthyroid middle aged male, and as the majority of patients presented in Table 1 he was, apart from the growing neck mass, without other clinical symptoms.

The diagnosis of primary thyroid lymphoma is sometimes challenging, with limited FNAC results, because of the histopathological similarities with Hashimoto thyroiditis [10] and anaplastic carcinoma [7, 30]. In clinically suspected cases it is suggested that diagnostics should be combined with flow cytometry [12, 31] even immunohistochemistry or molecular techniques including polymerase chain reaction (PCR) [12]. In our patient, the suspicion of PTL in the right lobe was made already on the basis of FNAC, but the final diagnosis of DLBC lymphoma, as well as of MALT lymphoma in the opposite lobe was made only after surgery and histopathological analysis as in the majority of cases reported in Table 1.

The diagnosis of chronic autoimmune lymphocytic thyroiditis is usually made on the basis of clinical presentation, laboratory findings (thyroid hormones, thyrotropin, and thyroid autoantibodies titre), thyroid ultrasound and sometimes FNAC. Hashimoto thyroiditis in our patient was an accidental histopathological finding, without any previous clinical suspicion.

The diagnosis of PTC usually includes ^99m^Tc pertechnetate scintigraphy where a “cold” nodule is found, ultrasound exam of the neck showing hypoechoic, but vascularized formation often with calcification, and finally FNAC of the nodule. In our case, although thyroid scintigraphy was not mandatory considering normal TSH value, it was performed to determine the functional status of nodules. Interestingly, PTC was not differentiated on US exam, probably because of the proximity of DLBCL lesion and small size, therefore it was only found on histopathological analysis.

The staging of lymphoma, as well as the assessment of therapy response, should be performed with ^18^F-FDG PET/CT, which has also been the case in our patient and the procedure did not document any disease spread. ^18^F-FDG PET/CT would be also indicated when dedifferentiation of papillary thyroid carcinoma is suspected.

The treatment of PTL includes chemotherapy according to R – CHOP protocol followed by conformal radiotherapy of the neck when remnant active disease is suspected. Although external radiotherapy could have been omitted in our patient, Onco-haematological team has decided in favour, considering the presence of metabolic activity on the first PET/CT scan.

The PTC treatment usually includes total thyroidectomy followed by RAI ablation of thyroid remnants with iodine-131, since PTC is well differentiated tumour with the ability to accumulate iodine, subsequently used in diagnostics and therapy. Upon completion of therapy, hormone supplementation with levothyroxine is indicated.

In our patient with multiple diagnoses, including DLBCL, MALT, PTC and HT, careful timing of therapeutic procedures was crucial. Initially, right lobectomy was performed due to Bethesda 5 report of the nodule. An 11 mm PTC was additionally found. Although in low-risk differentiated thyroid carcinoma total thyroidectomy is not obligatory, since high-level evidence is still lacking and intraglandular spread could not be excluded, the left sided lobectomy was additionally performed [32].

Otherwise, if only PTL was found, surgery would not be the method of choice [12, 33]. Since the success of the lymphoma therapy directly depends on the time interval from diagnosis, and considering very good prognosis of PTC, chemotherapy with subsequent neck irradiation for PTL was given a priority. Although recently published data suggest less aggressive therapeutic approach in low-risk differentiated thyroid carcinoma where RAI therapy could be omitted, it was administered to decrease the risk of disease recurrence and to eliminate thyroid tissue remnants, thus ensuring reliable follow-up through thyroglobulin measurements.

RAI therapy was, however, postponed allowing for the evaluation of the response to chemotherapy with control ^18^F-FDG PET/CT scan. After radioiodine ablation, supplementation therapy with levothyroxine was continued.

The prognosis of PTL depends on patient age and clinical stage, but it is generally more favourable for low grade lymphoma confined to thyroid gland, especially MALT lymphoma where the 5 years survival rate is almost 90%. However, for DLBCL type, 5 years survival rate is lower (75%) [34]. On the other hand, it has been very well known that 5 years survival for localised PTC is nearly 100%.

Our patient is on thyroid hormone supplementation and considered disease free for five years.

It can be concluded that in the case of known HT, periodical ultrasound of the neck is indicated. In the case of rapidly growing neck mass, besides anaplastic thyroid carcinoma, a suspicion on primary thyroid lymphoma should be raised. An optimal diagnostic and therapeutic management would include close collaboration between pathologist, cytologist, thyroid surgeon, haematologist and nuclear medicine specialist.

Since standard protocols for management of patients with multiple thyroid malignancies are missing, we consider that our case of simultaneous presence of MALT, DLBCL and PTC on Hashimoto background could add to the experience in order to correctly diagnose and treat these complex patients.

## Data Availability

Not applicable.
